# 1-Methyl-2-[(*E*)-2-(2-thien­yl)ethen­yl]quinolinium iodide^1^

**DOI:** 10.1107/S1600536808020734

**Published:** 2008-07-09

**Authors:** Pumsak Ruanwas, Thawanrat Kobkeatthawin, Suchada Chantrapromma, Hoong-Kun Fun, Chatchanok Karalai

**Affiliations:** a Crystal Materials Research Unit, Department of Chemistry, Faculty of Science, Prince of Songkla University, Hat-Yai, Songkhla 90112, Thailand; b X-ray Crystallography Unit, School of Physics, Universiti Sains Malaysia, 11800 USM, Penang, Malaysia

## Abstract

In the title compound, C_16_H_14_NS^+^·I^−^, the cation has an *E* configuration about the C=C double bond of the ethyl­ene unit. The dihedral angle between the thio­phene ring and the quinolinium ring system is 11.67 (11)°. A weak C—H⋯S intra­molecular inter­action involving the thio­phene ring generates an *S*(5) ring motif. In the crystal structure, the iodide ion, located between the cations arranged in an anti­parallel manner, forms weak C—H⋯I inter­actions. The crystal structure is further stabilized by a π–π inter­action between the thio­phene and pyridine rings; the centroid–centroid distance is 3.6818 (13) Å.

## Related literature

For bond lengths, see: Allen *et al.* (1987 ▶). For related literature on hydrogen-bond motifs, see: Bernstein *et al.* (1995 ▶). For related structures, see, for example: Chantrapromma *et al.* (2006 ▶, 2008 ▶); Chantrapromma, Jindawong & Fun (2007 ▶); Chantrapromma, Jindawong, Fun & Patil (2007 ▶). For background literature on non-linear optical properties, see, for example: Chou *et al.* (1996 ▶); Dittrich *et al.* (2003 ▶); Drost *et al.* (1995 ▶); Morley (1991 ▶).
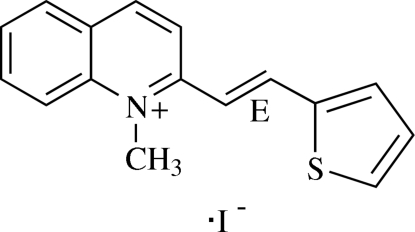


## Experimental

### 

#### Crystal data

C_16_H_14_NS^+^·I^−^
*M* *_r_* = 379.25
Triclinic, 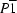

*a* = 7.8243 (1) Å
*b* = 9.6906 (1) Å
*c* = 10.7633 (2) Å
α = 97.521 (1)°
β = 95.338 (1)°
γ = 112.758 (1)°
*V* = 736.82 (2) Å^3^
*Z* = 2
Mo *K*α radiation
μ = 2.30 mm^−1^
*T* = 100.0 (1) K
0.58 × 0.28 × 0.14 mm

#### Data collection

Bruker SMART APEXII CCD area-detector diffractometer
Absorption correction: multi-scan (**SADABS**; Bruker, 2005 ▶) *T*_min_ = 0.346, *T*_max_ = 0.725
17060 measured reflections
4261 independent reflections
4118 reflections with *I* > 2σ(*I*)
*R*_int_ = 0.018

#### Refinement

*R*[*F* ^2^ > 2σ(*F* ^2^)] = 0.022
*wR*(*F* ^2^) = 0.059
*S* = 1.10
4261 reflections
173 parameters
H-atom parameters constrained
Δρ_max_ = 1.50 e Å^−3^
Δρ_min_ = −0.90 e Å^−3^

### 

Data collection: *APEX2* (Bruker, 2005 ▶); cell refinement: *APEX2*; data reduction: *SAINT* (Bruker, 2005 ▶); program(s) used to solve structure: *SHELXTL* (Sheldrick, 2008 ▶); program(s) used to refine structure: *SHELXTL*; molecular graphics: *SHELXTL*; software used to prepare material for publication: *SHELXTL* and *PLATON* (Spek, 2003 ▶).

## Supplementary Material

Crystal structure: contains datablocks global, I. DOI: 10.1107/S1600536808020734/is2311sup1.cif

Structure factors: contains datablocks I. DOI: 10.1107/S1600536808020734/is2311Isup2.hkl

Additional supplementary materials:  crystallographic information; 3D view; checkCIF report

## Figures and Tables

**Table 1:** Hydrogen-bond geometry (Å, °)

*D*—H⋯*A*	*D*—H	H⋯*A*	*D*⋯*A*	*D*—H⋯*A*
C10—H10*A*⋯S1	0.93	2.80	3.189 (2)	106
C11—H11*A*⋯I1^i^	0.93	3.06	3.934 (2)	157
C16—H16*B*⋯I1^ii^	0.96	3.06	3.962 (2)	156

Symmetry codes: (i) 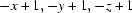; (ii) 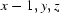.

**Fig. 1.**
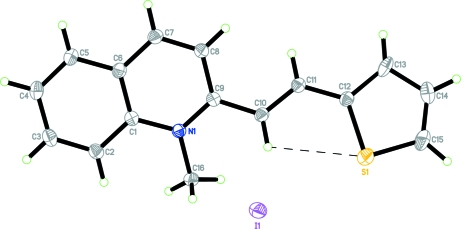
The title compound showing 50% probability displacement ellipsoids and the atom-numbering scheme. The weak C—H···S intramolecular interaction was drawn as a dashed line.

**Fig. 2.**
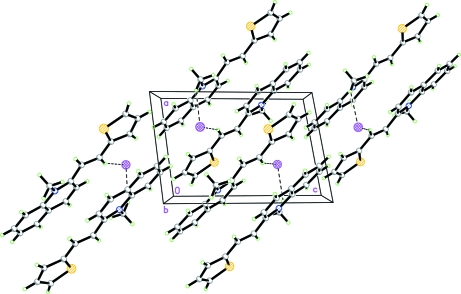
The packing diagram of the title structure, viewed approximately along the b axis. Weak C—H···I interactions were drawn as dashed lines.

## supplementary crystallographic information

### Comment

The design and synthesis of conjugated compounds to search for second-order nonlinear optic (NLO) materials have generated extensive interest. From previous reports, both molecular orbital calculations (Morley, 1991) and experimental studies (Drost *et al.*, 1995) have revealed that the products of dipole moment and molecular hyperpolarizability (υβ) of thiophene-containing conjugated moieties are superior to that of benzene analogues. Based on this reason we have previously studied the compound containing thiophene unit, namely, 1-methyl-4-[(*E*)-2-(2-thienyl)ethenyl]-pyridinium 4-chlorobenzenesulfonate (Chantrapromma *et al.*, 2008). In this paper we have synthesized the title compound which was designed by the replacement of the cationic 3-hydroxy-4-methoxyphenyl ring that is present in a compound possessing second-harmonic-generation (SHG) properties, 2-[(*E*)-2-(3-hydroxy-4-methoxyphenyl)ethenyl]-1methylquinolinium, iodide monohydrate (Chantrapromma, Jindawong, Fun & Patil, 2007) by the thiophene unit. Herein we report the synthesis and crystal structure of the title compound.

The asymmetric unit of the title compound (Fig. 1) consists of the C_16_H_14_NS^+^ cation and I^-^ ion. The cation exists in the *E* configuration with respect to the C10═C11 double bond [1.350 (3) Å] and is almost planar with the interplanar angle between the quinolinium and the thiophene ring being 11.67 (11)° and the torsion angles C9–C10–C11–C12 = -178.56 (17)°. The ethenyl unit is co-planar with the thiophene ring as can be indicated by the torsion angles C10–C11–C12–C13 = -179.42 (18)° and C10–C11–C12–S1 = 1.4 (3)°. It is slightly deviated from the quinolinium ring with the torsion angle C8–C9–C10–C11 = -14.2 (3)°. The atom S1 of the thiophene ring contributes to the C—H···S intramolecular weak interaction (Fig. 1 and Table 1) forming S(5) ring motifs (Bernstein *et al.*, 1995). The bond lengths and angles are normal (Allen *et al.*, 1987) and are comparable with closely related structures (Chantrapromma *et al.*, 2006, 2008; Chantrapromma, Jindawong & Fun, 2007; Chantrapromma, Jindawong, Fun & Patil, 2007).

In the crystal packing (Fig. 2), the I^-^ ion is in between each pair of the two antiparallel cations and is linked with the cations through weak C—H···I interactions. The crystal is stabilized by weak C—H···S and C—H···I interactions (Table 1). A π–π interaction was observed with the *Cg*_1_···*Cg*_2_ distance of 3.6818 (13) Å; *Cg*_1_^i^ and *Cg*_2_^i^ are the centroids of the S1/C12–C15 and N1/C1/C6–C9 rings, respectively [symmetry code: (i): 1 - *x*, 1 - *y*, 1 - *z*]. The perpendicular distances of *Cg*_2_ onto the plane of the S1/C12–C15 ring and *Cg*_1_ onto the plane of the N1/C1/C6–C9 ring are 3.200 and 3.500Å, respectively

### Experimental

2-(2-Thiophenestyryl)-1-methylquinilinium iodide was synthesized by mixing a solution (1:1:1 molar ratio) of 1,2-dimethylquinolinium iodide (2.00 g, 7.0 mmol), 2-thiophenecarboxaldehyde (0.64 ml, 7.0 mmol) and piperidine (0.69 ml, 7.0 mmol) in hot methanol (40 ml). The resulting solution was refluxed for 5 hr under a nitrogen atmosphere. The resultant solid was filtered off and washed with diethyl ether. Brown block-shaped single crystals of the title compound suitable for *x*-ray structure determination were obtained after recrystalization from methanol by slow evaporation of the solvent at room temperature after a few weeks.

### Refinement

All H atoms were placed in calculated positions (C—H = 0.93–0.96 Å) and were refined as riding, with *U*_iso_(H) = 1.2*U*_eq_(C) or 1.5*U*_eq_(methyl C). A rotating group model was used for the methyl group. The highest residual electron density peak is located at 0.75 Å from atom I1 and the deepest hole is located at 0.38 Å from atom S1.

### Crystal data

**Table:**

C_16_H_14_NS^+^·I^–^	*Z* = 2
*M**_r_* = 379.25	*F*_000_ = 372
Triclinic, *P*1	*D*_x_ = 1.709 Mg m^−^^3^
Hall symbol: -P 1	Mo *K*α radiation λ = 0.71073 Å
*a* = 7.8243 (1) Å	Cell parameters from 4261 reflections
*b* = 9.6906 (1) Å	θ = 2.3–30.0º
*c* = 10.7633 (2) Å	µ = 2.30 mm^−^^1^
α = 97.521 (1)º	*T* = 100.0 (1) K
β = 95.338 (1)º	Block, brown
γ = 112.758 (1)º	0.58 × 0.28 × 0.14 mm
*V* = 736.817 (18) Å^3^	

### Data collection

**Table:**

Bruker SMART APEXII CCD area-detector diffractometer	4261 independent reflections
Radiation source: fine-focus sealed tube	4118 reflections with *I* > 2σ(*I*)
Monochromator: graphite	*R*_int_ = 0.018
Detector resolution: 8.33 pixels mm^-1^	θ_max_ = 30.0º
*T* = 100.0(1) K	θ_min_ = 2.3º
ω scans	*h* = −11→11
Absorption correction: multi-scan(*SADABS*; Bruker, 2005)	*k* = −13→13
*T*_min_ = 0.346, *T*_max_ = 0.725	*l* = −15→15
17060 measured reflections	

### Refinement

**Table:**

Refinement on *F*^2^	Secondary atom site location: difference Fourier map
Least-squares matrix: full	Hydrogen site location: inferred from neighbouring sites
*R*[*F*^2^ > 2σ(*F*^2^)] = 0.022	H-atom parameters constrained
*wR*(*F*^2^) = 0.059	*w* = 1/[σ^2^(*F*_o_^2^) + (0.0282*P*)^2^ + 0.8519*P*] where *P* = (*F*_o_^2^ + 2*F*_c_^2^)/3
*S* = 1.10	(Δ/σ)_max_ = 0.002
4261 reflections	Δρ_max_ = 1.50 e Å^−^^3^
173 parameters	Δρ_min_ = −0.89 e Å^−^^3^
Primary atom site location: structure-invariant direct methods	Extinction correction: none

### Special details

**Table:**

Experimental. The low-temparture data was collected with the Oxford Cryosystem Cobra low-temperature attachment.
Geometry. All e.s.d.'s (except the e.s.d. in the dihedral angle between two l.s. planes) are estimated using the full covariance matrix. The cell e.s.d.'s are taken into account individually in the estimation of e.s.d.'s in distances, angles and torsion angles; correlations between e.s.d.'s in cell parameters are only used when they are defined by crystal symmetry. An approximate (isotropic) treatment of cell e.s.d.'s is used for estimating e.s.d.'s involving l.s. planes.
Refinement. Refinement of *F*^2^ against ALL reflections. The weighted *R*-factor *wR* and goodness of fit *S* are based on *F*^2^, conventional *R*-factors *R* are based on *F*, with *F* set to zero for negative *F*^2^. The threshold expression of *F*^2^ > σ(*F*^2^) is used only for calculating *R*-factors(gt) *etc*. and is not relevant to the choice of reflections for refinement. *R*-factors based on *F*^2^ are statistically about twice as large as those based on *F*, and *R*- factors based on ALL data will be even larger.

### Fractional atomic coordinates and isotropic or equivalent isotropic displacement parameters (Å^2^)

**Table:**

	*x*	*y*	*z*	*U*_iso_*/*U*_eq_	
I1	0.681003 (17)	0.790360 (14)	0.269777 (12)	0.02186 (5)	
S1	0.65928 (8)	0.89200 (6)	0.69144 (6)	0.02828 (11)	
N1	0.0896 (2)	0.54762 (17)	0.31586 (15)	0.0162 (3)	
C1	−0.0473 (3)	0.4405 (2)	0.21950 (17)	0.0167 (3)	
C2	−0.1289 (3)	0.4838 (2)	0.11749 (18)	0.0198 (3)	
H2A	−0.0945	0.5863	0.1138	0.024*	
C3	−0.2600 (3)	0.3734 (2)	0.02336 (19)	0.0227 (4)	
H3A	−0.3128	0.4025	−0.0441	0.027*	
C4	−0.3158 (3)	0.2180 (2)	0.02687 (19)	0.0227 (4)	
H4A	−0.4034	0.1452	−0.0382	0.027*	
C5	−0.2405 (3)	0.1739 (2)	0.12667 (19)	0.0206 (3)	
H5A	−0.2781	0.0711	0.1297	0.025*	
C6	−0.1062 (3)	0.2843 (2)	0.22486 (18)	0.0180 (3)	
C7	−0.0267 (3)	0.2412 (2)	0.32868 (18)	0.0193 (3)	
H7A	−0.0686	0.1391	0.3357	0.023*	
C8	0.1112 (3)	0.3491 (2)	0.41844 (18)	0.0180 (3)	
H8A	0.1644	0.3198	0.4855	0.022*	
C9	0.1745 (2)	0.5058 (2)	0.41052 (17)	0.0161 (3)	
C10	0.3282 (3)	0.6203 (2)	0.50081 (18)	0.0175 (3)	
H10A	0.3838	0.7174	0.4822	0.021*	
C11	0.3950 (3)	0.5929 (2)	0.61089 (17)	0.0174 (3)	
H11A	0.3399	0.4946	0.6273	0.021*	
C12	0.5444 (3)	0.7036 (2)	0.70463 (18)	0.0177 (3)	
C13	0.6158 (3)	0.6721 (2)	0.82202 (19)	0.0217 (4)	
H13A	0.5752	0.5776	0.8468	0.026*	
C14	0.7577 (3)	0.8091 (3)	0.8930 (2)	0.0282 (4)	
H14A	0.8203	0.8145	0.9724	0.034*	
C15	0.7946 (3)	0.9321 (3)	0.8350 (2)	0.0300 (4)	
H15A	0.8850	1.0285	0.8706	0.036*	
C16	0.1413 (3)	0.7112 (2)	0.31385 (19)	0.0211 (3)	
H16A	0.1810	0.7677	0.3991	0.032*	
H16B	0.0345	0.7250	0.2756	0.032*	
H16C	0.2418	0.7471	0.2655	0.032*	

### Atomic displacement parameters (Å^2^)

**Table:**

	*U*^11^	*U*^22^	*U*^33^	*U*^12^	*U*^13^	*U*^23^
I1	0.02044 (7)	0.02041 (7)	0.02481 (7)	0.00758 (5)	0.00089 (5)	0.00842 (5)
S1	0.0287 (3)	0.0207 (2)	0.0312 (3)	0.00775 (19)	−0.0017 (2)	0.00210 (19)
N1	0.0151 (7)	0.0161 (7)	0.0176 (7)	0.0066 (5)	0.0021 (5)	0.0033 (5)
C1	0.0158 (7)	0.0191 (8)	0.0165 (8)	0.0083 (6)	0.0035 (6)	0.0031 (6)
C2	0.0179 (8)	0.0227 (9)	0.0192 (8)	0.0082 (7)	0.0029 (6)	0.0058 (7)
C3	0.0202 (8)	0.0295 (10)	0.0184 (8)	0.0101 (7)	0.0013 (7)	0.0056 (7)
C4	0.0178 (8)	0.0262 (9)	0.0197 (8)	0.0065 (7)	−0.0009 (7)	−0.0006 (7)
C5	0.0188 (8)	0.0193 (8)	0.0213 (8)	0.0068 (7)	0.0007 (7)	−0.0005 (7)
C6	0.0161 (8)	0.0187 (8)	0.0184 (8)	0.0068 (6)	0.0023 (6)	0.0019 (6)
C7	0.0195 (8)	0.0165 (8)	0.0217 (8)	0.0075 (7)	0.0028 (7)	0.0028 (6)
C8	0.0183 (8)	0.0177 (8)	0.0181 (8)	0.0077 (6)	0.0017 (6)	0.0033 (6)
C9	0.0153 (7)	0.0175 (8)	0.0164 (7)	0.0074 (6)	0.0036 (6)	0.0030 (6)
C10	0.0178 (8)	0.0154 (7)	0.0188 (8)	0.0064 (6)	0.0023 (6)	0.0021 (6)
C11	0.0158 (8)	0.0173 (8)	0.0190 (8)	0.0070 (6)	0.0026 (6)	0.0023 (6)
C12	0.0168 (8)	0.0163 (8)	0.0198 (8)	0.0069 (6)	0.0026 (6)	0.0015 (6)
C13	0.0121 (7)	0.0217 (9)	0.0238 (9)	0.0014 (6)	0.0068 (6)	−0.0071 (7)
C14	0.0231 (9)	0.0395 (12)	0.0201 (9)	0.0132 (9)	−0.0010 (7)	0.0002 (8)
C15	0.0263 (10)	0.0248 (10)	0.0295 (11)	0.0056 (8)	−0.0030 (8)	−0.0066 (8)
C16	0.0219 (9)	0.0163 (8)	0.0245 (9)	0.0075 (7)	−0.0003 (7)	0.0051 (7)

### Geometric parameters (Å, °)

**Table:**

S1—C15	1.697 (2)	C7—H7A	0.9300
S1—C12	1.7273 (19)	C8—C9	1.421 (2)
N1—C9	1.354 (2)	C8—H8A	0.9300
N1—C1	1.397 (2)	C9—C10	1.446 (3)
N1—C16	1.481 (2)	C10—C11	1.350 (3)
C1—C2	1.410 (3)	C10—H10A	0.9300
C1—C6	1.413 (3)	C11—C12	1.436 (3)
C2—C3	1.377 (3)	C11—H11A	0.9300
C2—H2A	0.9300	C12—C13	1.450 (3)
C3—C4	1.403 (3)	C13—C14	1.420 (3)
C3—H3A	0.9300	C13—H13A	0.9300
C4—C5	1.371 (3)	C14—C15	1.361 (4)
C4—H4A	0.9300	C14—H14A	0.9300
C5—C6	1.412 (3)	C15—H15A	0.9300
C5—H5A	0.9300	C16—H16A	0.9600
C6—C7	1.415 (3)	C16—H16B	0.9600
C7—C8	1.364 (3)	C16—H16C	0.9600
			
C15—S1—C12	91.58 (11)	N1—C9—C8	119.08 (16)
C9—N1—C1	121.89 (16)	N1—C9—C10	119.79 (16)
C9—N1—C16	119.57 (16)	C8—C9—C10	121.13 (17)
C1—N1—C16	118.53 (15)	C11—C10—C9	123.13 (17)
N1—C1—C2	121.92 (17)	C11—C10—H10A	118.4
N1—C1—C6	118.98 (16)	C9—C10—H10A	118.4
C2—C1—C6	119.09 (17)	C10—C11—C12	125.11 (17)
C3—C2—C1	119.54 (18)	C10—C11—H11A	117.4
C3—C2—H2A	120.2	C12—C11—H11A	117.4
C1—C2—H2A	120.2	C11—C12—C13	124.49 (17)
C2—C3—C4	121.53 (19)	C11—C12—S1	123.74 (15)
C2—C3—H3A	119.2	C13—C12—S1	111.77 (14)
C4—C3—H3A	119.2	C14—C13—C12	108.83 (19)
C5—C4—C3	119.69 (18)	C14—C13—H13A	125.6
C5—C4—H4A	120.2	C12—C13—H13A	125.6
C3—C4—H4A	120.2	C15—C14—C13	114.4 (2)
C4—C5—C6	120.20 (18)	C15—C14—H14A	122.8
C4—C5—H5A	119.9	C13—C14—H14A	122.8
C6—C5—H5A	119.9	C14—C15—S1	113.38 (17)
C5—C6—C1	119.91 (18)	C14—C15—H15A	123.3
C5—C6—C7	121.09 (17)	S1—C15—H15A	123.3
C1—C6—C7	118.99 (17)	N1—C16—H16A	109.5
C8—C7—C6	120.12 (17)	N1—C16—H16B	109.5
C8—C7—H7A	119.9	H16A—C16—H16B	109.5
C6—C7—H7A	119.9	N1—C16—H16C	109.5
C7—C8—C9	120.69 (18)	H16A—C16—H16C	109.5
C7—C8—H8A	119.7	H16B—C16—H16C	109.5
C9—C8—H8A	119.7		
			
C9—N1—C1—C2	−176.75 (17)	C1—N1—C9—C8	−5.6 (3)
C16—N1—C1—C2	3.6 (3)	C16—N1—C9—C8	173.99 (16)
C9—N1—C1—C6	3.5 (3)	C1—N1—C9—C10	173.83 (16)
C16—N1—C1—C6	−176.05 (16)	C16—N1—C9—C10	−6.6 (2)
N1—C1—C2—C3	178.41 (17)	C7—C8—C9—N1	3.2 (3)
C6—C1—C2—C3	−1.9 (3)	C7—C8—C9—C10	−176.23 (17)
C1—C2—C3—C4	0.5 (3)	N1—C9—C10—C11	166.39 (17)
C2—C3—C4—C5	0.8 (3)	C8—C9—C10—C11	−14.2 (3)
C3—C4—C5—C6	−0.6 (3)	C9—C10—C11—C12	−178.56 (17)
C4—C5—C6—C1	−0.8 (3)	C10—C11—C12—C13	−179.42 (18)
C4—C5—C6—C7	−179.96 (18)	C10—C11—C12—S1	1.4 (3)
N1—C1—C6—C5	−178.25 (16)	C15—S1—C12—C11	178.33 (17)
C2—C1—C6—C5	2.0 (3)	C15—S1—C12—C13	−0.98 (15)
N1—C1—C6—C7	0.9 (3)	C11—C12—C13—C14	−177.85 (18)
C2—C1—C6—C7	−178.77 (17)	S1—C12—C13—C14	1.5 (2)
C5—C6—C7—C8	175.96 (18)	C12—C13—C14—C15	−1.3 (3)
C1—C6—C7—C8	−3.2 (3)	C13—C14—C15—S1	0.6 (3)
C6—C7—C8—C9	1.2 (3)	C12—S1—C15—C14	0.22 (19)

### Hydrogen-bond geometry (Å, °)

**Table:**

*D*—H···*A*	*D*—H	H···*A*	*D*···*A*	*D*—H···*A*
C10—H10A···S1	0.93	2.80	3.189 (2)	106
C11—H11A···I1^i^	0.93	3.06	3.934 (2)	157
C16—H16B···I1^ii^	0.96	3.06	3.962 (2)	156

Symmetry codes:  (i) −*x*+1, −*y*+1, −*z*+1; (ii) *x*−1, *y*, *z*.
